# Netrin-1 stimulated axon growth requires the polyglutamylase TTLL1

**DOI:** 10.3389/fnins.2024.1436312

**Published:** 2024-10-14

**Authors:** Kyle R. Northington, Jasmynn Calderon, Emily A. Bates

**Affiliations:** Department of Pediatrics, University of Colorado Anschutz Medical Campus, Aurora, CO, United States

**Keywords:** microtubule polyglutamylation, microtubule-associated protein 1B, DCX = doublecortin, Netrin-1, axon growth and guidance, tubulin (microtubules), TTLL1

## Abstract

**Introduction:**

In the developing brain, neurons extend an axonal process through a complex and changing environment to form synaptic connections with the correct targets in response to extracellular cues. Microtubule and actin filaments provide mechanical support and drive axon growth in the correct direction. The axonal cytoskeleton responds to extracellular guidance cues. Netrin-1 is a multifunctional guidance cue that can induce alternate responses based on the bound receptor. The mechanism by which actin responds to Netrin-1 is well described. However, how Netrin-1 influences the microtubule cytoskeleton is less understood. Appropriate microtubule function is required for axon pathfinding, as mutations in tubulin phenocopy axon crossing defects of Netrin-1 and DCC mutants. Microtubule stabilization is required for attractive guidance cue response. The C-terminal tails of microtubules can be post-translationally modified. Post-translational modifications (PTMs) help control the microtubule cytoskeleton.

**Methods:**

We measured polyglutamylation in cultured primary mouse cortical neurons before and after Netrin-1 stimulation. We used immunohistochemistry to measure how Netrin-1 stimulation alters microtubule-associated protein localization. Next, we manipulated TTLL1 to determine if Netrin-1-induced axon growth and MAP localization depend on polyglutamylation levels.

**Results:**

In this study, we investigated if Netrin-1 signaling alters microtubule PTMs in the axon. We found that microtubule polyglutamylation increases after Netrin-1 stimulation. This change in polyglutamylation is necessary for Netrin-1-induced axonal growth rate increases. We next determined that MAP1B and DCX localization changes in response to Netrin-1. These proteins can both stabilize the microtubule cytoskeleton and may be responsible for Netrin-1-induced growth response in neurons. The changes in DCX and MAP1B depend on TTLL1, a protein responsible for microtubule polyglutamylation.

## Introduction

Axon pathfinding is a vital process that paves the way for neuronal circuit formation during brain development. Failure in axon pathfinding can lead to connectivity defects and age-related neurodegeneration (Buscaglia et al., 2021; Wegiel et al., 2018; Rachel et al., 2000; Livesey and Hunt, 1997). Netrin-1 is a well-established guidance cue that controls a bundle of axons crossing the midline of the brain called the corpus callosum and commissures (Yung et al., 2015; Bin et al., 2015). Netrin-1 knockout mice fail to form commissures or the corpus callosum (Fothergill et al., 2014). Netrin-1 knockout axons form disorganized bundles at either side of the midline known as Probst bundles (Fothergill et al., 2014).

Netrin-1 stimulates axon growth by binding its receptor Deleted in Colorectal Cancer (DCC) (Buscaglia et al., 2021; Hill et al., 2012; Varadarajan and Butler, 2017; Dent, 2004; Shekarabi and Kennedy, 2002; Li et al., 2002). Like Netrin-1 deletion, DCC knockout mice fail to form commissures indicating that Netrin-1 signaling through DCC is essential for axon guidance across the midline (Yung et al., 2015; Fothergill et al., 2014). In addition, axonal response to Netrin-1 depends upon the microtubule cytoskeleton (Buscaglia et al., 2021; Gasperini et al., 2017; Piper et al., 2015). Microtubules are dynamic polymers of *α*-and *β*-tubulin heterodimers that undergo periods of growth and depolymerization. The dynamic instability of microtubules helps drive axon extension or retraction in response to a guidance cue. Agenesis or hyperplasia of the corpus callosum and other commissures is associated with mutations that disrupt neuronally expressed tubulin in humans and mice indicating that microtubules are important for axon guidance in response to Netrin-1 (Buscaglia et al., 2021; Gartz Hanson et al., 2016; Bahi-Buisson et al., 2014; Bahi-Buisson and Maillard, 1993; Aiken et al., 2017). Microtubule stabilization is also required for axon response to guidance cues (Piper et al., 2015; Buck and Zheng, 2002). However, the mechanisms underlying how Netrin-1 affects microtubule properties are not understood.

Post-translational modifications (PTMs) may be a mechanism to rapidly change microtuble properties in response to guidance cues. PTMs to tubulin can alter microtubule properties and affect the binding of certain microtubule-associated proteins (MAPs) (Verhey and Gaertig, 2007; Chakraborti et al., 2016; Janke and Magiera, 2020). PTMs and MAPs regulate the stability of the microtubule polymer and affect axon growth (Dema et al., 2024; Friocourt et al., 2003; Jean et al., 2012; Bonnet et al., 2001; Jentzsch et al., 2024; Xu et al., 2017; Portran et al., 2017). Tubulin PTMs provide temporal and spatial control along the microtubule by altering MAP binding, kinesin activity, and intrinsic tubulin interactions (Bonnet et al., 2001; Xu et al., 2017; Portran et al., 2017; Marcos et al., 2009; Utreras et al., 2008; Lessard et al., 2019). The addition of glutamate residues to the *α*-and *β*-tubulin carboxy-terminal tails (polyglutamylation) alters the localized charge of the microtubule lattice (Janke and Magiera, 2020; Janke, 2014; Janke et al., 2008; Audebert et al., 1993; Bodakuntla et al., 2021; Bodakuntla et al., 2021; Ruse et al., 2022). Therefore, microtubule polyglutamylation changes the binding activity of specific MAPs and alters the trafficking of motor proteins to affect axonal growth (Friocourt et al., 2003; Bonnet et al., 2001; Bigman and Levy, 2020; Bodakuntla et al., 2020). This raises the possibility that Netrin-1 may regulate microtubule dynamics via altering polyglutamylation. In neurons, microtubule polyglutamylation is important for neuronal survival and function (Bodakuntla et al., 2021; Bodakuntla et al., 2020; Bedoni et al., 2016; Magiera et al., 2018; Shashi et al., 2018; Wang and Morgan, 2007). Furthermore, microtubule polyglutamylation affects MAP binding and motor trafficking rates, which could affect axon growth (Bonnet et al., 2001; Lessard et al., 2019; Bigman and Levy, 2020; Bodakuntla et al., 2020)., This demonstrates that polyglutamylation levels can regulate microtubule networks. Additionally, microtubule polyglutamylation levels are rapidly tuned in cells (Torrino et al., 2021), on a similar timescale to changes in axon length in response to Netrin-1. Together, these data support the premise that polyglutamylation could regulate the microtubule cytoskeleton for axon response to axon guidance cues like Netrin-1.

Polyglutamylation is controlled by Tubulin Tyrosine Ligase Like (TTLL) proteins that add glutamate residues to tubulin heterodimers and cytosolic carboxypeptidase (CCP) proteins that remove glutamate residues (Janke et al., 2005). TTLL proteins can initiate the branch point glutamate residue or elongate a glutamate chain. TTLL1 extends glutamate chains on *α*-and *β*-tubulin (Ping et al., 2023; Wu et al., 2022). TTLL1 is highly expressed in the brain (Janke et al., 2005). TTLL1 is necessary and sufficient to increase microtubule polyglutamylation in neurons, suggesting that TTLL1 polyglutamylates microtubules in neurons (Bodakuntla et al., 2020). Together, these data form the premise for the hypothesis that polyglutamylation may regulate microtubule response to guidance cues.

In this study, we show Netrin-1 increases microtubule polyglutamylation in the axon. We show that TTLL1 is required for axonal response to Netrin-1. We should that two important MAPs, MAP1b and DCX, localize to the axon in response to Netrin-1. Finally, we show that the localization of MAP1B and DCX to the axon in response to Netrin-1 depends upon TTLL1. These data suggest that TTLL1 is important for axonal response to Netrin-1.

## Materials and methods

### Animal care

C57Bl6 (RRID:IMSR_JAX:000664) mice were housed in pathogen-free facilities approved by AALAC. Procedures were performed under protocol 139 approved by the IACUC at The University of Colorado, Anschutz Medical Campus. Mice were kept on a 14:10 h light:dark cycle with *ad libitum* access to food and water. Mice were set up in breeding pairs. Pups were taken between postnatal day (P) 0 and P4 for all experiments.

### Primary cortical neuron dissections, nucleofection, and culture

For primary neuronal cultures, mice were taken between P0 and P4 for dissection. The head was sprayed down with 70% ethanol and a decapitation was performed. The brain was removed and placed on a plate containing Hanks Balanced Salt Solution (Gibco Cat# 14175095) with 200 mL kynurenic acid, referred to as Dissection Media (DM). The hindbrain was resected. The brain was split along the midline and the meninges was removed. Next, the cortex was isolated and split into small pieces. These pieces were placed into a conical containing 3 mL of DM. Cortical pieces were then moved to a conical containing DM supplemented with papain, L-cysteine, and kynurenic acid. The conical was placed into a 37°C incubator for 45 min. After 45 min the papain solution was aspirated and replaced with 4 mL of plating media containing DMEM with glucose and sodium pyruvate, Glutamax, and pen/strep. Cells were resuspended and then allowed to settle before the media was aspirated again. Fresh plating media was added to the cells. Using a narrow bore Pasteur pipette, the cells were triturated between 10 and 20 times. This process breaks down all the pieces into a single-cell suspension. Neurons were then spun down at 400 RCF for 5 min. Media was aspirated and the cells were resuspended for downstream processes. **Nucleofection:** Primary cortical neurons were co-nucleofected with equal volumes of marker plasmids and a different plasmid of interest. For example, 4 μg of GFP-CSAP (Dr. Chad Pearson, CU Anschutz) and 4 μg of myrTdTomato (Dr. Santos Franco, CU Anschutz) were added together to *Nucleofector Solution for Mouse Neurons with Supplement 1* (Lonza Cat# VPG-1001) in the same tube to create lipid droplets with both plasmids. For experiments manipulating TTLL1, neurons were nucleofected with 4 μg of TTLL1 OE plasmid or 4 μg TTLL1 shRNA plasmid along with 4 μg of a plasmid expressing a fluorescence marker (either myrTdTomato or GFP-CSAP). The solution was mixed by pipetting. Primary neurons were centrofuged and media was removed. Cells were resuspended in 50 μL of *Nucleofector solution with Supplement 1* and 50 μL of the plasmid/ Nucleofector solution before transfer to the nucleofection cuvette. Neurons were nucleofected using the O-03 setting on the Lonza Nucleofector 2b (Lonza Cat# 13458999). Nucleofected cells recovered in 2 mL of culture media with additional L-glutamine supplementation for 30 min in a 37°C incubator. Neurons were then plated for growth overnight in a 37°C incubator. After 24 h in culture, neurons were imaged on a Zeiss 900 microscope. 24 h after plating, the media was replaced with Neurobasal A without phenol red supplemented with B-27, 1X Glutamax, and b-FGF for all experiments.

### Netrin-1 production and purification

Using an established protocol for Netrin-1 purification, Cos-7 cells were transfected with a Netrin-1 plasmid (OriGene Cat#: MG223704) using Lipofectamine 3,000 (Thermo Fisher Scientific Cat#: L3000015) (McCormick et al., 2024; Mutalik et al., 2024; Boyer et al., 2020; Plooster et al., 2017). Cells were incubated at 37°C with 5% CO_2_ overnight. The next day, DMEM was removed, and cells were washed twice with PBS. OptiMEM serum-free media was added, and the cells were incubated for 24 additional hours. Next, the OptiMEM was removed and placed into a conical. The conical was spun down at 1400xg for 3 min to remove debris and dead cells. The media was then moved to a calibrated Amicon 30 kDa molecular weight cutoff centrifuge tube (Cat#: UFC903008). The tube was spun down at 3000xg at 4°C for 5 min and the flow-through was discarded. The tube was spun down for another 5 min and the flow-through was again discarded. Additional 1 min spins were performed until the filter portion of the tube contained ~500 μL of media. This was then removed and used for downstream experiments. The protein was then run on an SDS gel and stained with Coomassie blue to ensure the appropriate-sized band (80 kDa) was detected (Supplementary Figure S1). A BCA assay (Pierce) was used run to determine the concentration of the protein.

### Western blots

Cortical neurons were dissected from mice between P0 and P3. Cells were plated in a 6-well plate coated with poly-d-lysine. Cultured primary neurons were exposed to 500 ng/mL of Netrin-1. Netrin-1 was left on the cells for either 5, 10, or 20 min before the media was removed. Cells were washed once with 2 mL of PBS to remove excess media. PBS was removed, 2 mL of fresh PBS was added, and neurons were scraped off the bottom of the dish using a cell scraper. Cells were spun down and PBS was removed. Cells were resuspended in RIPA buffer containing protease and phosphatase inhibitors. Protein abundance was determined using the Pierce BCA assay. Afterward, Lameli buffer was added to the samples. Western blots were performed using BioRad 4–20% gels and run at 65 V for 2 to 3 h. Protein was transferred using the BioRad Trans Blot Turbo system. Blots were washed in 1X TBS and then blocked for 1 h in 5% milk in 1X TBST. Primary antibodies were added and were left to incubate overnight on a shaker at 4°C. Primary antibodies were removed, and the membrane was washed with 1X TBST 3 times for 5 min each time. Secondary antibodies were diluted in 5% milk in 1X TBST and added to the membrane. The membrane was placed on a shaker for 1 h at room temperature. Secondary antibodies were removed, and the blot was washed with 1X TBST for 5 min 3 times. BioRad ECL developer was added to the blot for 5 min and left on a shaker before imaging of the blot was performed. All blots were imaged using a BioRad imaging system. Densitometry was analyzed using FIJI. Polyglutamylation levels were normalized to the amount of GAPDH protein expression seen on the blot. A ratio of polyglutamylation to GAPDH was used to determine the change in expression before and after the addition of Netrin-1.

### Neuron growth rate experiments

Primary cortical neurons were nucleofected with 4 mg of MyrTdTomato. The neurons were plated and cultured for 24 h. Images were taken on a Zeiss 900 confocal microscope with a 20X air objective. Images were captured every 10 min before and every 10 min after 500 ng/mL of Netrin-1 was added to the media. Images were analyzed in FIJI using the line segment tool. Lengths were measured from the beginning of the axon to the longest tip of the growth cone. The change in length between each time point was calculated and graphed as DLength.

### GFP-CSAP imaging

Primary cortical neurons were nucleofected with 4 μg of GFP-CSAP (Dr. Chad Pearson, CU Anschutz) and 4 μg of MyrTdTomato (Dr. Santos Franco, CU Anschutz). After 24 h in culture, neurons were imaged on a Zeiss 900 microscope. For additional GFP-CSAP experiments neurons were nucleofected with 4 μg of TTLL1 OE plasmid, TTLL1 shRNA, or scramble control plasmid. Images were taken every 10 min for 30 min before adding Netrin-1 at 500 ng/mL. Images were taken immediately and every 10 min for 30 min after Netrin-1 was added. Images were analyzed in FIJI, where a threshold was set and maintained individually per neuron and kept across every time point. ROIs were taken at 5 μm away from the soma, 20 μm away from the soma, 5 μm away from the growth cone and the growth cone for normal GFP-CSAP loacalization changes. For GFP-CSAP data collected in the Supplementary Figures the entire axon was measured as we previously observed changes in GFP-CSAP along multiple points of the axon.

### Neuron morphology analysis

Neurons were nucleofected with 4 μg of MyrTdTomato and 4 μg of TTLL1 overexpression plasmid or scramble shRNA control. The neurons were plated and cultured for 24 h. Images were taken on a Zeiss 900 confocal microscope with a 20X air objective. Neurons were analyzed using a Scholl analysis plugin with FIJI. Images were cropped to include only the axon within the image. Primary branch points from the axon were counted and compared between the TTLL1 OE neurons and MyrTdTomato expressing neurons.

### Immunofluorescence

Each mouse cortex was dissociated into single neurons which were divided between 8 wells on a cover slip (ThermoFisher product #177402). Plates were removed from the incubator to room temperature and 500 ng/mL Netrin-1 or an equal volume of vehicle was added. After either 5, 10, 20, or 30 min of Netrin-1 or vehicle exposure, media was aspirated and the cells were washed with PBS. Cells were fixed with a solution of 4% PFA and 0.1% glutaraldehyde in PBS for 10 min at room temperature. The fixation solution was removed, and cells were washed with PBS. Cells were then washed using 3% BSA with 0.2% Triton-X in PBS for 5 min. Next, cells were washed with a solution of 0.1% NaBH_4_ in PBS for 7 min at room temperature on a shaker. The reducing buffer was removed and cells were washed 3 times with PBS for 5 min. Cells were blocked in 3% BSA with 0.2% Triton-X in PBS for 20 min at room temperature on a shaker. The blocking buffer was removed and primary antibodies were added and left overnight at 4°C on a shaker. The primary antibody was removed, and cells were washed with 0.2% BSA and 0.05% Triton-X in PBS 3 times for 10 min. Secondary antibodies were added and placed in the dark at room temperature for 30 min on a shaker. Cells were then washed with 0.2% BSA and 0.05% Triton-X in PBS 3 times for 10 min. One additional wash was performed with PBS. A coverslip was then placed on the slide along with Fluromount-G with DAPI. Slides were stored at 4°C in the dark until imaging was performed. Images were taken on a Zeiss 900 confocal microscope. Images were analyzed in FIJI. A threshold was set to eliminate background fluorescence and the cells were measured with one ROI containing the soma, one containing the entire axon, and one containing the growth cone. Mean fluorescence intensity is reported. MAP1B and DCX fluorescence intensity were compared between Netrin-1-treated and vehicle-treated controls at 5 and 10 min after exposure to account for any change in MAP1B or DCX that occurs due to mechanical force of liquid addition or time at room temperature. Statistics were performed in GraphPad Prism 10. Student’s *t*-tests were performed between groups.

### Statistical analyses

Statistics were performed in GraphPad Prism 10. Statistical significance was reported as a *p*-value of <0.05. Specific statistical analyses are reported in each figure legend. For all graphs mean ± SEM is shown unless otherwise noted. In comparisons between two groups a Student’s *t*-test was performed.

To account for photobleaching of GFP-CSAP that occurred when imaging TTLL1 shRNA and scramble control neurons, we assessed the rate of decay before and after the addition of Netrin-1 in both samples. We estimate the following equation:


logyit=αi+β1timet+β2timet×postt+β3postt+β4timet×ttll1i+β5postt×ttll1i+β6timet×postt×ttll1i+εit'

which measures the log of fluorescence for neuron *i* at minute t and represents an idiosyncratic error term. We allow for neuron-specific fluorescence with individual fixed effects (αi). We model the fluorescence decay allowing it to differ by ttll1shRNA both before and after the addition of Netrin-1, such that β_1_ captures the decay rate for the control group before Netrin-1 is added, *β*_2_ measures the change in the decay rate for the control after Netrin-1 is added, and *β*_3_ measures any level shift in log fluorescence with the addition of Netrin-1. We measure the difference of each these measures for the ttll1 shRNA sample coefficients *β*_4_, *β*_5_, and *β*_6_ respectively, paying particular attention to *β*_6_, the difference between the ttll1 shRNA sample and the control sample in the change in the decay rate after adding Netrin-1. We cluster our standard errors by neuron so that our inference is robust to autocorrelation within the same neuron over time. While the rate of decay was exponential before Netrin-1 was added to both samples, it flattened after the addition of Netrin-1 in the control group but continued to decay in the treatment group.

**Table 1:** 

**Dissection Media**
HBSS (Ca_2+_ and Mg_2+_ free) Cat. # 14170112
1 M HEPES Cat. # 15630106
Kynurenate solution
**Plating Media**
DMEM w/ glucose and sodium pyruvate Cat. # 11995065
Glutamax (100X) Cat. # 35050061
Pen/Strep (100X) Cat. # 15070063
**Maintenance Media**
Neurobasal A Cat. # 12349015
B27 (50X) Cat. # 17504001
Glutamax (100X) Cat. # 35050061
B-FGF (0.1 mg/mL) Cat. # 450–33-100UG
**Borate Buffer**
Boric Acid Cat. # B6768-500G
Sodium tetraborate
MilliQ Water (pH 8.5)
**Plate Coating**
Borate Buffer
Poly-D Lysine Stock Cat. # A3890401
**Kynurenate Solution**
Kynurenic Acid
10N NaOH Cat. # SS255-1
MilliQ Water
**Papain Solution**
HBSS (Ca_2+_ and Mg_2+_ free) Cat. # 14170112
Kynurenate (100 mM)
Papain
Cysteine (1 M)
DNAse I Cat. # 11284932001
**Plasmids**
MACF43-GFP
MyrTdTomato (Dr. Santos Franco, CU Anschutz)
GFP-CSAP (Dr. Chad Pearson, CU Anschutz)
Netrin-1 OE OriGene Cat#: MG223704
TTLL1 OE OriGene Cat#: NM_178869
TTLL1 shRNA Santa Cruz Biotech Cat#: sc-154786-SH
Control shRNA plasmid Santa Cruz Biotech Cat#:sc-108060
**Antibodies**
Rabbit Polyglutamylated Tubulin AdipoGen Cat#: AG-25B-0030-C050
Rabbit GAPDH Cell Signaling Technologies Cat#:2118
Goat Doublecortin Invitrogen Cat#: PA5-142704
Mouse MAP1B Santa Cruz Biotech Cat#: sc-135978
Rabbit Total Beta Tubulin: Invitrogen Cat # PA1-16947
Mouse Alpha Tubulin DM1A Sigma Cat #T6199
anti-Polyglutamylation Modification, mAb (GT335) AdipoGen Cat# AG-20B-0020-C100
Goat Anti-Rabbit IgG (H + L) Alexa Fluor Plus 555 Invitrogen Cat#:A32732
Goat Anti-Mouse Invitrogen Cat#:A11001
Donkey Anti-Goat IgG (H + L) Alexa Fluor Plus 647 Invitrogen Cat#:A32849
VECTASHIELD Vibrance Antifade Mounting Medium with DAPI Vector Laboratories Cat#:H-1800
Donkey Anti-Rabbit HRP Santa Cruz Biotech Cat#:sc-2313
Goat Anti-Mouse HRP Santa Cruz Biotech Cat#:sc-2005
Precision Protein StrepTactin-HRP Conjugate Bio-Rad Cat#:1610380

## Results

### Netrin-1 alters microtubule polyglutamylation along the axon

**Figure 1:**
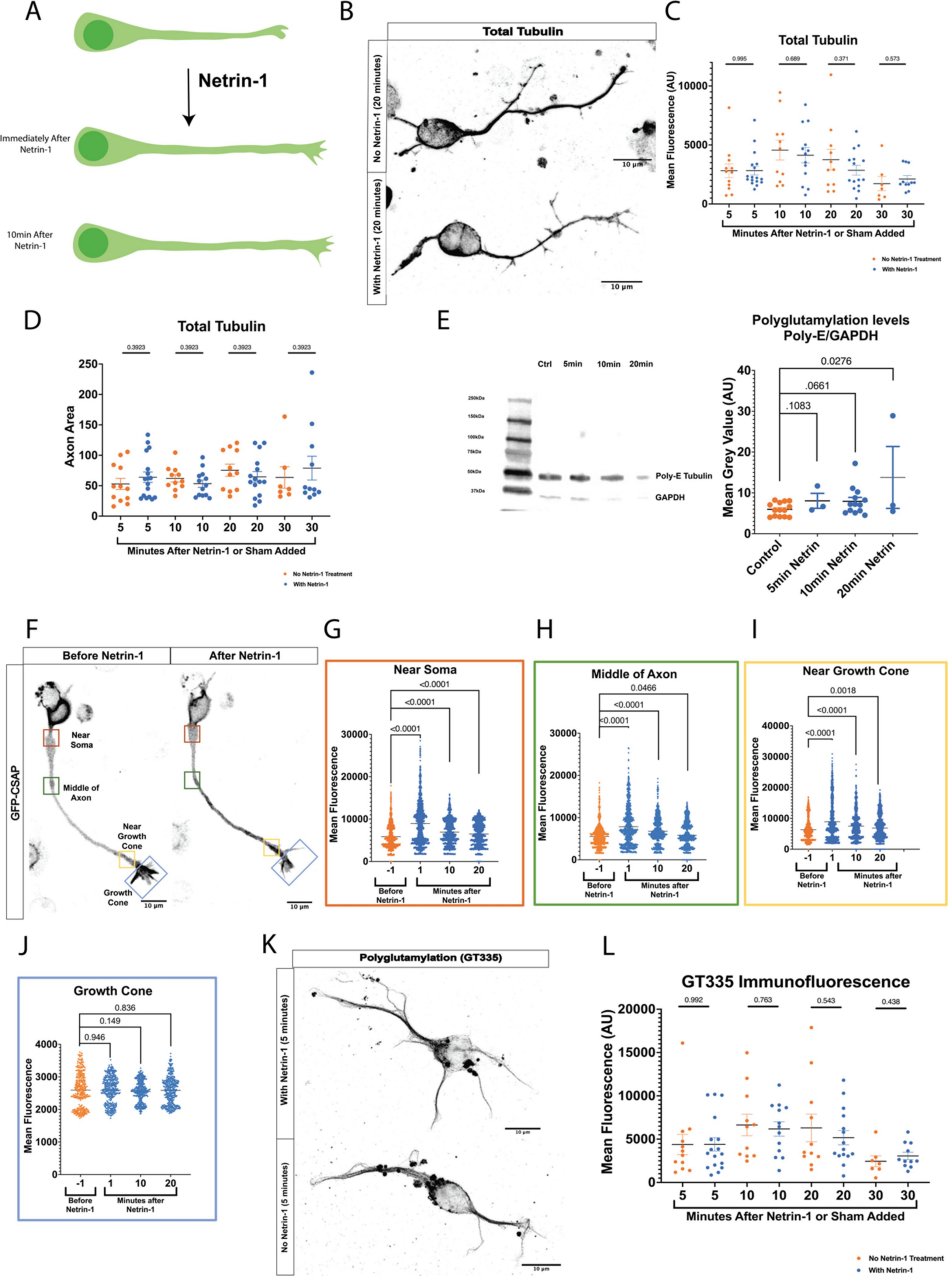
**(A)** Netrin-1 causes an increase in axon growth rate. However, it is unknown how the microtubule cytoskeleton is regulated to allow for this increase in growth rate. **(B)** Representative images of neurons stained for total tubulin. **(C)** Fluorescence data from total tubulin stained neurons shows no increase in total tubulin levels following Netrin-1 stimulation (*N* = cortical neurons from 6 mice). **(D)** Representative Western Blots and quantified densitometry show that polyglutamylation/GAPDH increases after Netrin-1 stimulation. *N* = cultured cortical neurons from 6 mice for no Netrin-1 and 10 min Netrin-1, *N* = cultured cortical neurons from 3 mice for 5 min Netrin-1, and 20 min Netrin-1. **(E)** Representative image showing example locations along the neuron where ROIs were selected. **(F)** Near Soma shows an increase in CSAP fluorescence intensity following Netrin-1 stimulation (*N* = 23 CSAP expressing cortical neurons from 8 mice). **(G)** The Middle of the Axon also experiences an increase in CSAP fluorescence intensity following Netrin-1 stimulation. **(H)** Near the Growth Cone also shows an increase in CSAP fluorescence intensity after Netrin-1 stimulation. **(I)** The Growth Cone showed no differences in GFP CSAP at any time following Netrin-1 addition to the media. **(J)** Representative images of neurons stained with the glutamylated tubulin antibody GT335 which mark glutamylated tubulin independent of glutamate chain length. **(K)** Quantified levels of GT335 immunofluorescence glutamylated tubulin show no increase in levels following Netrin-1 stimulation (*N* = cortical neurons from 6 mice).

Netrin-1 increases axonal growth rate rapidly (Figure 1A) (Buscaglia et al., 2021). Axons could respond to Netrin-1 by increasing total polymerized tubulin or modifying established microtubules. We tested whether total tubulin levels in the axon increase in response to Netrin-1 by measuring total tubulin immunofluorescence and area in mouse primary cultured cortical neurons before and after exposure Netrin-1. Tubulin immunofluorescence did not increase after Netrin-1 exposure (Figures 1B,C). Furthermore, the total area of axonal tubulin fluorescence did not increase between neurons exposed to Netrin-1 and unexposed cultured neurons (Figure 1D). We hypothesized that neurons increase PTM abundance along the microtubule in response to Netrin-1. We quantified levels of polyglutamylation before and after Netrin-1 stimulation in primary cultured cortical neurons with western blots. Polyglutamylated tubulin normalized to GAPDH levels increased within 20 min following Netrin-1 stimulation and trended towards an increase after 10 min (Figure 1E). Centriole and Spindle-Associated Protein (CSAP) localizes to polyglutamylated microtubules (Bompard et al., 2018; Backer et al., 2012). For spatial and temporal resolution to visualize where and when polyglutamylation levels change with Netrin-1 stimulation, we used GFP-CSAP as a live polyglutamylation reporter and measured fluorescence intensity at multiple locations along the axon (Figures 1F–J; Supplementary Video S1) (Backer et al., 2012). Netrin-1 stimulation significantly increased the fluorescence intensity of GFP-CSAP in the axon shaft immediately following its application and for up to 20 min afterward (Figures 1F–I). There was no significant change in GFP-CSAP intensity in the growth cone at any point following Netrin-1 stimulation (Figure 1J). To distinguish between whether neurons increase the *initiation* of glutamylation or *extension* of glutamylated chains on tubulin tails in the axon in response to Netrin-1, we measured immunofluorescence of the GT335 glutamylation antibody, which recognizes the first two glutamates on the tubulin carboxy terminal tail, with and without Netrin-1 exposure. Immunofluorescence of GT335 in the axon did not increase with Netrin-1 exposure (Figures 1K,L) indicating that Netrin-1 does not induce new initiation of glutamylation chains on the tubulin tail. Rather, Netrin-1 stimulation increases the abundance of long glutamate side chains with 4 or more glutamate residues in the axon (Figure 1C). These data indicate that MTs are dynamically altered through post-translational modifications in response to Netrin-1. We next probed whether microtubule polyglutamylation is required for the axon growth rate increase in response to Netrin-1.

### TTLL1 is required for axon growth response to Netrin-1

**Figure 2:**
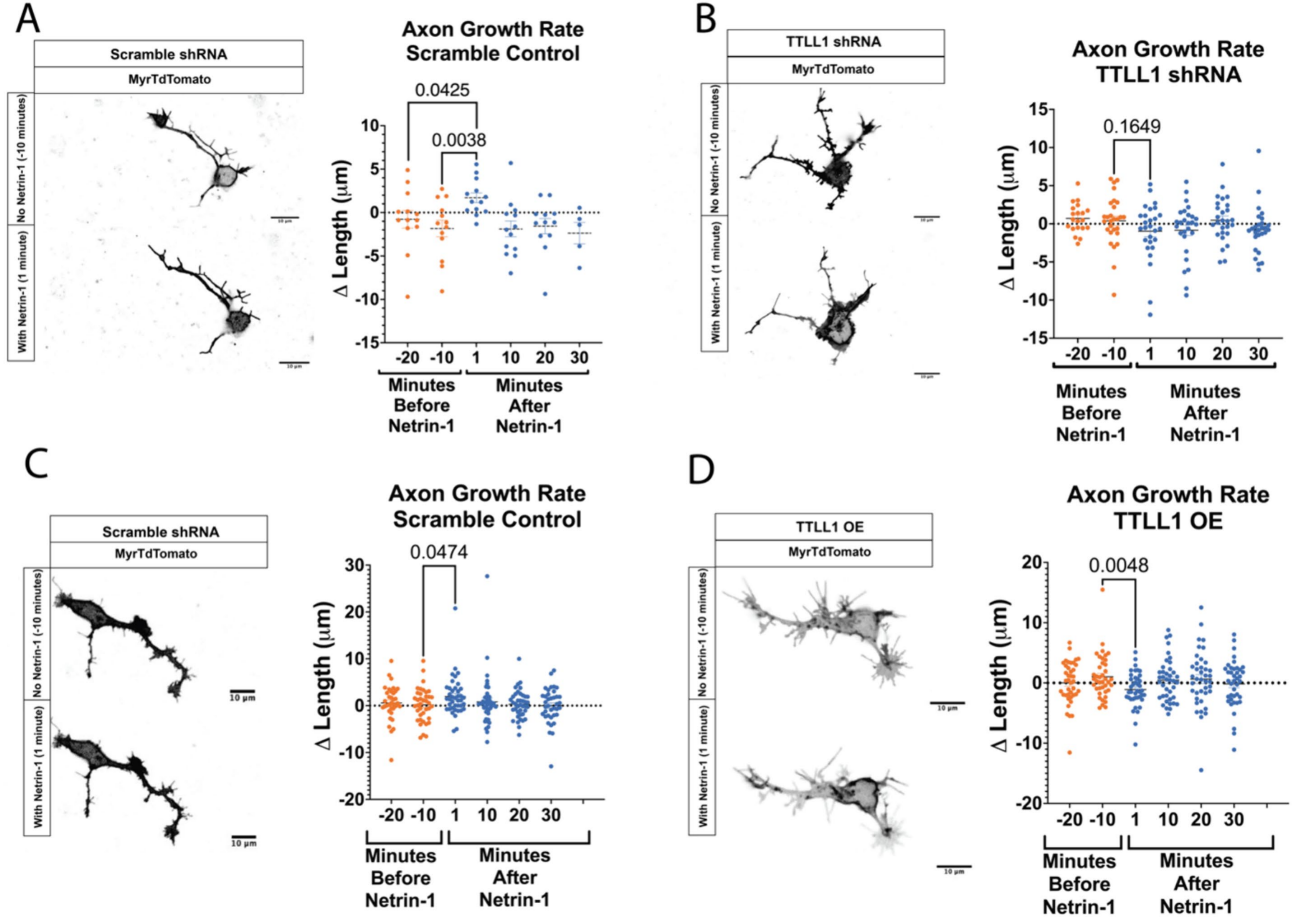
Growth rate following Netrin-1 stimulation is TTLL1 dependent. **(A)** Axons expressing a scramble shRNA that are time matched to those shown in B can respond to Netrin-1 exposure by increasing their growth rate (*N* = 5 or more neurons from 2 mice). **(B)** Axon growth no longer increases after Netrin-1 exposure in TTLL1 shRNA neurons (*N* = At least 20 neurons from 3 mice). **(C)** In neurons expressing a scramble shRNA performed at the same time as those in D experience an increase in growth rate following Netrin-1 exposure (*N* = at least 35 neurons from 3 mice). **(D)** In TTLL1 OE neurons, there is a significant decrease in the growth rate following Netrin-1 exposure (*N* = 40 neurons from 3 mice).

We hypothesized that precise control of polyglutamylation regulates microtubule stability to promote Netrin-induced growth response. TTLL1 extends polyglutamylation chains on the tubulin carboxy-terminal tails in neurons, while other TTLLs are responsible for initiation (Trichet et al., 2000; Wang et al., 2022). To test whether TTLL1 was required for the increase in axonal polyglutamylation in response to Netrin-1, we performed live imaging of neurons co-nucleofected with GFP-CSAP and TTLL1 shRNA plasmid. CSAP fluorescence decayed rapidly due to photobleaching even after addition of Netrin-1 in TTLL1 shRNA expressing neurons, while CSAP fluorescence flattens with the addition of Netrin-1 in scramble controls (Supplementary Figure S2). These data suggest that TTLL1 is required to increase polyglutamylation in response to Netrin-1. To determine if TTLL1 is required for axon growth in response to Netrin-1, we reduced TTLL1 expression in primary cortical neurons with TTLL1 shRNA and measured axon growth rate using a membrane-bound TdTomato protein before and after Netrin-1 stimulation. We measured the change in length from the soma to the most distal tip of the growth cone over time in TTLL1 shRNA and the scramble control. We observed that similar to previously published data (Buscaglia et al., 2021), control neurites increased in growth rate following Netrin-1 exposure (Figure 2A). TTLL1 knockdown abolished changes in neurite growth rate following the addition of Netrin-1 (Figure 2B).

We reasoned that polyglutamylation could be sufficient for an increase in growth rate. We overexpressed TTLL1 and measured CSAP fluorescence before and after Netrin-1. CSAP fluorescence does not increase in response to Netrin-1 in neurons that overexpress TTLL1 (Supplementary Figure S2). These data suggest that microtubule polyglutamylation does not increase in response to Netrin-1 in TTLL1 overexpressing neurons. To determine if a change in microtubule polyglutamylation is required for an increase in growth rate, we overexpressed TTLL1 in primary cortical neurons and measured neurite growth response to Netrin-1. Whereas control neurons increase in response to Netrin-1, TTLL1 overexpression significantly decreased neurite growth rate following the addition of Netrin-1 (Figures 2C,D). An abundance of TTLL1 inhibits neurite growth response to Netrin-1. These data support the hypothesis that TTLL1 is required to increase microtubule polyglutamylation for neurite growth response to Netrin-1. We observed that TTLL1 overexpression changes neuronal morphology. A Scholl analysis showed that TTLL1 overexpressing neurons significantly increases the number of branch points along the axon (Supplementary Figure S3). Altering the levels of TTLL1 in either direction is detrimental to axon response to Netrin-1 stimulation. We next wanted to determine whether MAPs that stabilize microtubules increase localization to the axon in response to Netrin-1.

### Netrin-1 stimulation increases MAP abundance

**Figure 3:**
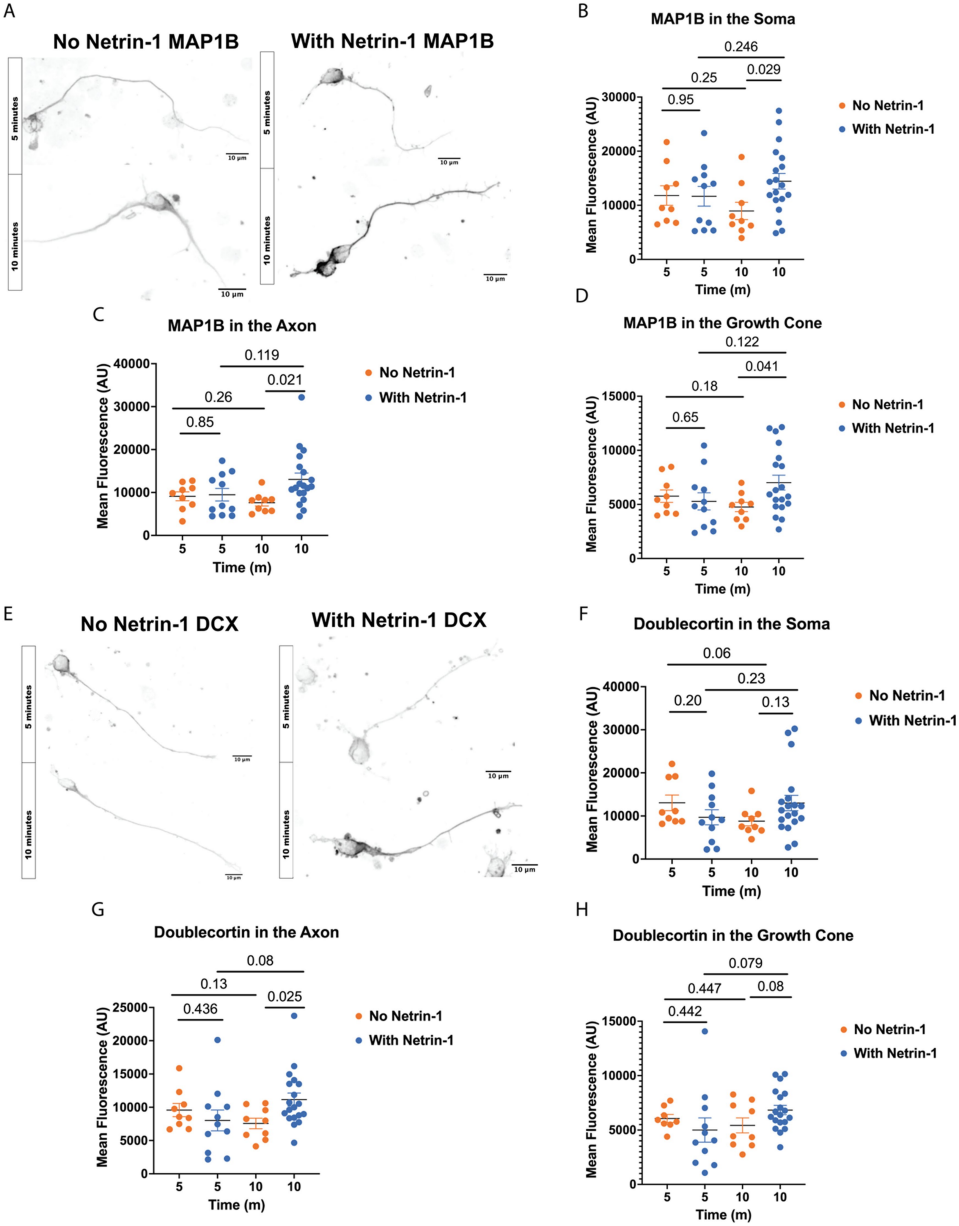
Netrin-1 stimulation changes MAP localization. **(A)** Representative images of MAP1B in neurons at DIV1 after fixation, Scale Bar 10 μm. **(B)** MAP1B in the soma changes with Netrin-1 after 10 min (*N* = 9 or more neurons from 2 animals). **(C)** MAP1B changes in the axon after 10 min of Netrin-1 stimulation (*N* = 9 or more neurons from 2 animals). **(D)** MAP1B increases in the growth cone following Netrin-1 stimulation. **(E)** Representative images of DCX in DIV1 neurons after fixation, Scale Bar 10 μm. **(F)** Doublecortin does not change in the soma following Netrin-1 stimulation (*N* = 9 or more neurons from 2 animals). **(G)** Doublecortin does increase in the axon following Netrin-1 stimulation for 10 min (*N* = 9 or more neurons from 2 animals). **(H)** Doublecortin trends towards an increase in the growth cone following 10 min of Netrin-1 stimulation (*N* = 9 or more neurons from 2 animals).

Microtubule polyglutamylation alters the charge of the C-terminal tail thereby changing the binding affinity of certain MAPs for the microtubule surface. MAP1B is an essential MAP required for Netrin-1 signaling and commissure formation (del Río et al., 2004; Jayachandran et al., 2016; Meixner et al., 2000; Shi et al., 2019; Takei et al., 2000). To test if MAP1B localization changes with Netrin-1 stimulation, we fixed and stained neurons for MAP1B at multiple times after Netrin-1 or vehicle addition (Figures 3A–D). Netrin-1 addition significantly increases MAP1B fluorescence intensity in the soma, along the axon, and in the growth cone after 10 min of exposure (Figures 3B–D), while there is a trend towards a decrease in MAP1B fluorescence 10 min after vehicle addition.

Doublecortin (DCX) may be important for the axon response to guidance cues (Dema et al., 2024; Sébastien et al., 2023; Tint et al., 2009). To determine if DCX localization changes in response to Netrin-1, we stained primary neurons with Netrin-1 for DCX in a time course after stimulation with Netrin-1 (Figure 3E). Netrin-1 exposure significantly increases DCX in the axon (Figure 3G). DCX trends towards increasing in the growth cone after Netrin-1, but does not alter DCX in the soma (Figures 3F–H). These results indicate that neurons increase the localization of DCX to the axon in response to Netrin-1. The increase in DCX and MAP1B fluorescence intensity occurs after the period when GFP-CSAP fluorescence increases following Netrin-1 stimulation (Figure 1), suggesting that increasing polyglutamylation levels may recruit or aid in trafficking MAP1B and DCX. Is Netrin-1-induced localization of MAP1B and DCX dependent on precise control of TTLL1 levels?

### TTLL1 overexpression changes MAP localization in response to Netrin-1

**Figure 4:**
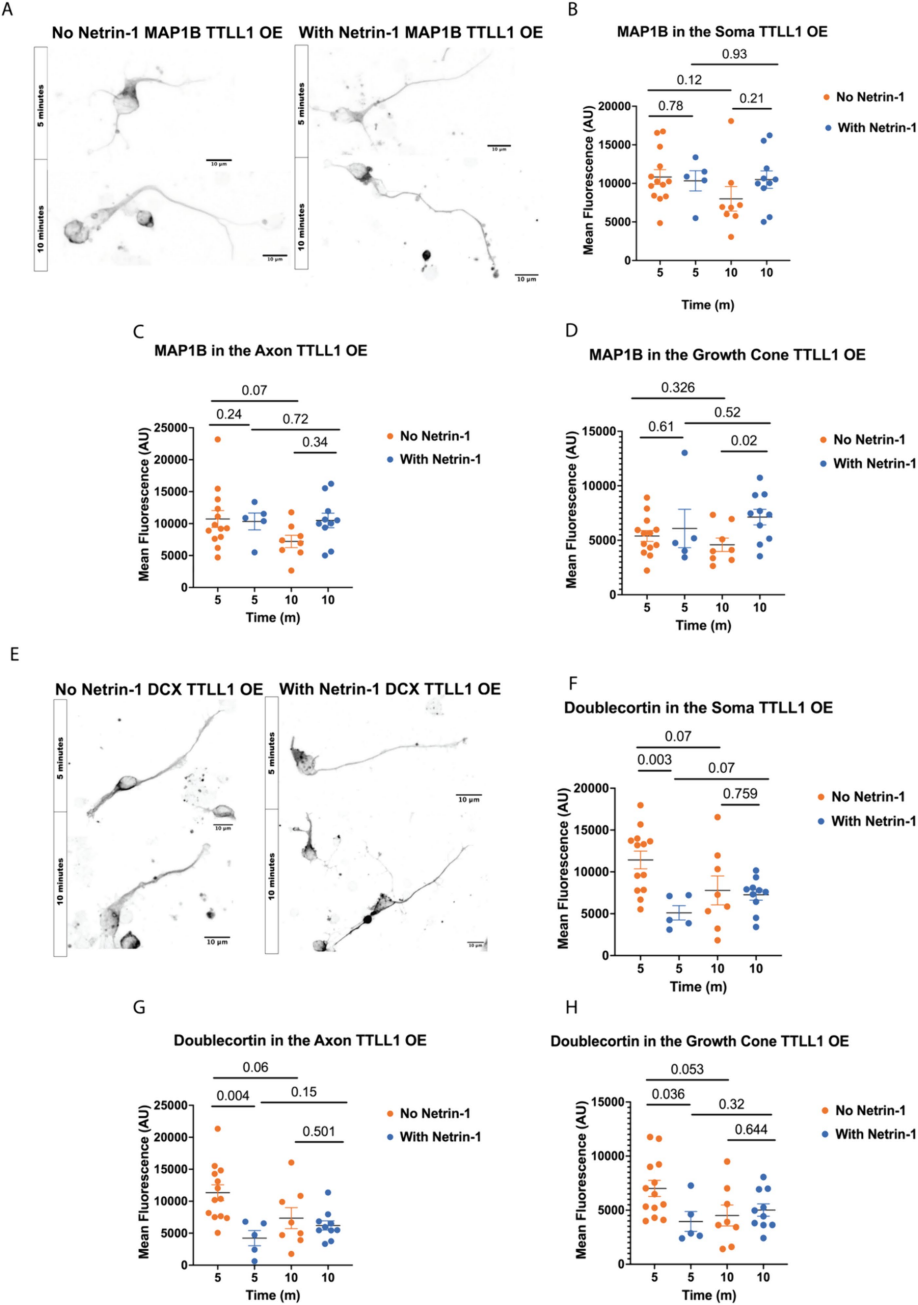
TTLL1 OE alters Netrin-1 induced changes in MAP localization. **(A)** Representative images of MAP1B in TTLL1 OE neurons at DIV1 after fixation, Scale Bar 10 μm. **(B)** MAP1B in the soma does not change with Netrin-1 after 10 min (*N* = Minimum of 5 neurons from 2 animals). **(C)** MAP1B does not change in the axon after 10 min of Netrin-1 stimulation (*N* = Minimum of 5 neurons from 2 animals). **(D)** MAP1B continues to increase in the growth cone following Netrin-1 stimulation in TTLL1 OE neurons (*N* = Minimum of 5 neurons from 2 animals). **(E)** Representative images of DCX in DIV1 TTLL1 OE neurons after fixation, Scale Bar 10 μm. **(F)** Doublecortin does not change in the soma following Netrin-1 stimulation (*N* = Minimum of 5 neurons from 2 animals). **(G)** Doublecortin does not increase in the axon following Netrin-1 stimulation for 10 min (*N* = Minimum of 5 neurons from 2 animals). **(H)** Doublecortin does not increase in the growth cone following 10 min of Netrin-1 stimulation (*N* = Minimum of 5 neurons from 2 animals).

To determine whether precise microtubule polyglutamylation regulation is necessary for MAP localization in response to Netrin-1, we overexpressed TTLL1 in cultured primary cortical neurons, stimulated them with Netrin-1, and stained neurons for MAP1B and DCX (Figures 4A,E). MAP1B staining does not increase in the axon after 10 min of Netrin-1 exposure in TTLL1 OE neurons as it does in control neurons (Figure 4C). TTLL1 overexpression prevents Netrin-1-induced MAP1B increases in the soma (Figure 4B). However, MAP1B still increases in the growth cone of TTLL1 OE neurons (Figure 4D). In TTLL1 OE neurons, DCX fluorescence in the axon, soma, and growth cone is reduced 5 min after Netrin-1 exposure compared to vehicle-exposed neurons at the same time point. There were no changes in DCX fluorescence 10 min following Netrin-1 exposure in any area measured in cortical neurons overexpressing TTLL1 compared to vehicle exposed neurons (Figures 4F–H). TTLL1 overexpression prevents Netrin-1-induced increases in polyglutamylation, MAP1B, and DCX localization to the axon, and neurite growth rate. Together, these data show that Netrin-1-induced MAP1B and DCX localization to the axon are dependent on TTLL1.

## Discussion

### Regulation of polyglutamylation is important for axon response to Netrin-1

**Figure 5:**
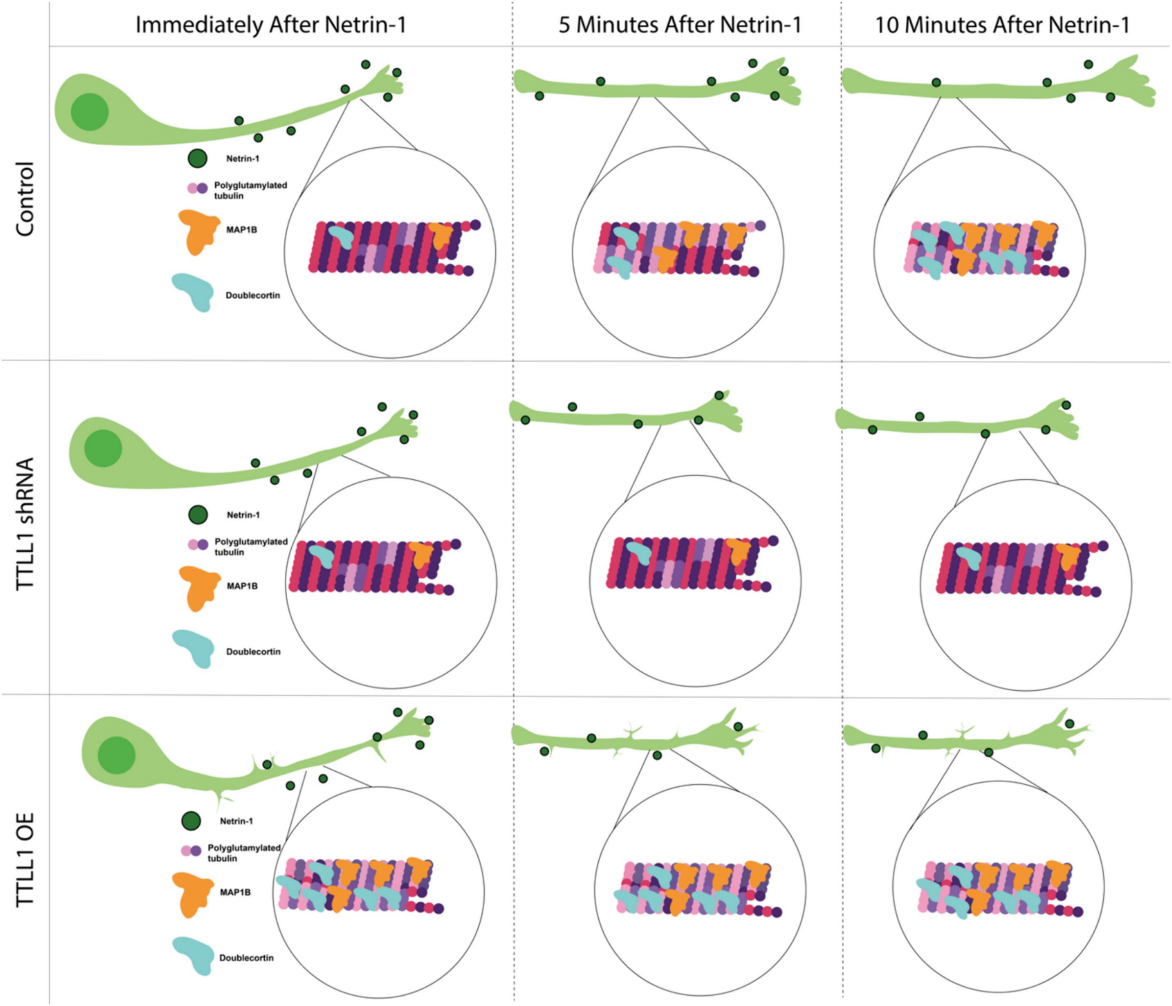
Model mechanism showing Netrin-1 stimulation increases polyglutamylation of microtubules over time. This increased polyglutamylation leads to increases in MAP1B and DCX in the axon, which stabilizes the microtubule cytoskeleton. This increased stability allows for improved axon growth following Netrin-1 stimulation.

The mechanism by which Netrin-1 communicates with the microtubule cytoskeleton has been a significant knowledge gap. Here, we show that microtubule polyglutamylation increases in response to Netrin-1 (Figure 1), and that increased polyglutamylation is required for the axon growth response to Netrin-1 (Figure 2). Both MAP1B and DCX increase in abundance along the axon in response to Netrin-1 (Figure 3). The increase in axon growth rate may be due to a stabilizing effect from MAP1B or DCX (Figures 3, 4). The localization changes of MAP1B and DCX in the axon require regulated TTLL1 activity (Figure 4). Our data supports the model that Netrin-1 stimulation rapidly increases TTLL1 activity to promote microtubule polyglutamylation. Polyglutamylation changes the microtubule charge to promote the binding of stabilizing MAPs such as MAP1B and DCX (Figure 5). We propose that the increase in microtubule polyglutamylation and MAP binding stabilizes the lattice, promoting increased axon growth (Figure 5).

### Post-translational modifications

Post-translational modifications can regulate microtubule function in a myriad of ways, The increase in polyglutamylation along the axon after Netrin-1 exposure may regulate microtubule response to external stimuli. Previous work in the field has shown changes in microtubule polyglutamylation in response to external mechanical forces (Torrino et al., 2021). We see increases in GFP-CSAP fluorescence in response to Netrin-1 a minute after the chemotactic cue is added to the media and it stays elevated for at least 20 min (Figure 1). However, in western blot analysis, we do not see significant increases in polyglutamylation until 20 min after the addition of Netrin-1 (Figure 1). Additionally, the changes seen in polyglutamylation in the western blot are likely long glutamate chains, as Netrin-1 does not increase the immunofluorescence of GT335 glutamylation antibody, which marks the initial two glutamates added to the tubulin C-terminal tail (Figure 1). While we use GFP-CSAP as a proxy for polyglutamylation, it may also play a role in changing the polyglutamylation state of microtubules (Bompard et al., 2018). That confounding factor is a limitation of our study. Further studies could investigate how PTMs respond to guidance cues using nanobodies which would enable live-imaging with the necessary protein specificity (Barakat et al., 2022; Freise and Wu, 2015; Fu et al., 2018).

Netrin-1 stimulation increases microtubule polyglutamylation in the axon through the action of TTLL1. Netrin-1-stimulated DCC could directly or indirectly activate TTLL1. Netrin-1-induced increases in polyglutamylation are too rapid for transcription or translation of new TTLL1. One possibility is that Netrin-1 stimulation increases glutamate available to TTLL1 to add to microtubules or other substrates. Glutamine metabolism to generate glutamate is required for microtubule polyglutamylation (Torrino et al., 2021). Reducing available glutamate reduces microtubule stability suggesting that polyglutamylation increases microtubule stability directly or indirectly through changing affinity for MAPs (Torrino et al., 2021). Glutamate levels also regulate axon growth (Schmitz et al., 2009; Zheng et al., 1996; Kreibich et al., 2004). For example, glutamate stimulates axon growth in cultured dopaminergic neurons (Schmitz et al., 2009). Furthermore, cultured spinal cord neurites turn towards a glutamate source (Zheng et al., 1996). While we have thought of glutamate stimulating axonal growth through calcium, glutamate availability could be a limiting reagent for its addition to microtubules. How polyglutamylation-modifying enzyme activity is modulated in the context of Netrin-1 will be an exciting area of future research.

Extensive research has defined mechanisms by which Netrin-1 stimulates changes in the actin cytoskeleton for axon guidance (McCormick et al., 2024; Mutalik et al., 2024; Boyer et al., 2020; Plooster et al., 2017; Menon et al., 2021; Menon et al., 2015). Co-immunoprecipitations or BioID experiments could shed light on the mechanism by which Netrin-1 bound DCC stimulates TTLL1 activity. It is also a possibility that intermediate pathways facilitate signaling between the DCC receptor and the microtubule cytoskeleton. However, DCC interacts with *β*-tubulin and this may allow for nearby tubulin modifying enzymes to alter microtubule PTMs (Qu et al., 2013). This raises the possibility that there could be a complex including TTLLs and DCC to modify the microtubule cytoskeleton in response to receptor activation. This could be validated through future BioID or Co-IP experiments.

This study focused on polyglutamylation; however, numerous post-translational modifications can occur on the microtubule lattice. How additional microtubule modifications are altered in response to guidance cues is an intriguing area of future research that could deepen our understanding of cytoskeletal regulation during development. The tyrosination/detyrosination cycle is an interesting candidate for further study as it regulates pathfinding (Marcos et al., 2009). Additionally, MAP1B interacts with Tubulin Tyrosine Ligase protein which controls tubulin tyrosination (Utreras et al., 2008). Post-translational modifications of tubulin during axon guidance remain an exciting area of research. Microtubule PTMs can alter intrinsic lattice dynamics and how MAPs and motors bind.

Polyglutamylation recruits spastin, a microtubule severing enzyme that Is an important regulator of microtubule dynamics (Lacroix et al., 2010; Valenstein and Roll-Mecak, 2016). Interestingly, spastin breaks the microtubule lattice and increases local microtubule polymerization to regulate synapse formation (Aiken and Holzbaur, 2024). An increase in spastin activity causes branching in neurons (Yu et al., 2008). The increased axon branching phenotype observed in TTLL1 OE neurons may be due to increased spastin activity acting on hyper-glutamylated microtubules (Supplementary Figure S3). There is also the possibility that these branches are actin-mediated, as Netrin-1 has long been associated with changes in actin cytoskeleton regulation (Shekarabi and Kennedy, 2002; Li et al., 2002; Menon et al., 2021; Shekarabi et al., 2005; Boyer and Gupton, 2018). PTM control of microtubule properties continues to be an area of active research. Our study offers some insight into how microtubules are regulated in developing neurons. These results indicate important changes to polyglutamylation occur *in vitro* and in the specific cells that perform these migrations.

### Microtubule-associated proteins in the developing brain

Microtubule-associated proteins offer another layer of regulation of the microtubule lattice. The variety of MAP functions can provide precise regional control over the stability and function of microtubules. We show that MAP1B fluorescence increases ten minutes after addition of Netrin-1, while it trends towards decreasing ten minutes after addition of vehicle. The trend towards a decrease in MAP1B in vehicle exposed neurons could be due to temperature changes or consequences of mechanical stimulation with the addition of media. Polyglutamylation increases microtubule stability and is localized to axons and growth cones (Bonnet et al., 2001; Lessard et al., 2019). MAP1B is required for axon response to Netrin-1 and stabilizes the microtubule cytoskeleton in neurites (del Río et al., 2004; Meixner et al., 2000; Li et al., 2006). MAP1B may preferentially bind to polyglutamylated microtubules (Bonnet et al., 2001) and this could be the mechanism through which Netrin-1 signaling promotes microtubule stability. Altering TTLL enzyme levels may change glutamate chain length, which could regulate the affinity of MAP1B for the microtubule lattice. The increase in MAP1B axonal localization in response to Netrin-1 could stabilize the microtubule lattice and allow for increased axon growth in response to Netrin-1 (Figure 2). The increase in GFP-CSAP and MAP1B in the axon following Netrin-1 are supports the model that Netrin-1 increases polyglutamylation which recruits MAP1B or aids in its localization to the axon. TTLL1 OE abolishes the increase in axon growth and MAP1B localization to the axon following Netrin-1 stimulation, supports the model that TTLL1 is required for axon growth and MAP1B localization to the axon. However, our results in the growth cone are not consistent with this model. While Netrin-1 does not measurably increase GFP-CSAP in the growth cone, Netrin-1 increases MAP1B in the growth cone (Figures 1J, 3D). Furthermore, Netrin-1 increases MAP1B in the growth cone of TTLL1 OE neurons (Figure 4D). These data indicate that MAP1B localization is either not dependent on polyglutamylation in the growth cone, or that GFP-CSAP does not localize to the growth cone adequately to assess changes in polyglutamylation. Another possibility is that overexpressing TTLL1 does not affect polyglutamylation in the growth cone.

We report an increase of DCX fluorescence in the axon ten minutes after Netrin-1 stimulation, while we observe a trend towards a decrease in DCX fluorescence ten minutes after addition of vehicle (Figure 3). We observe a trend towards an increase in DCX fluorescence in the growth cone after Netrin-1 stimulation. This raises the possibility that microtubule stability in the axon, behind the growth cone, is important for overall response to Netrin-1. DCX knockout mice have reduced polyglutamylation levels and fail to respond to guidance cues (Dema et al., 2024; Sébastien et al., 2023). DCX localizes to microtubules in the growth cone in a highly polarized fashion and stabilizes microtubule polymer (Dema et al., 2024; Friocourt et al., 2003). Neuronal DCX knockouts fail to respond to brain-derived neurotrophic factor gradients indicating an important role for DCX in axon guidance (Dema et al., 2024). Specific levels of microtubule polyglutamylation recruit spastin, a MAP that regulates axonal microtubule dynamics in specific localizations to facilitate appropriate axonal transport (Lacroix et al., 2010; Valenstein and Roll-Mecak, 2016). DCX is also important for actin response to Netrin-1 through its effect on actin-binding proteins, suggesting another mechanism by which polyglutamylation could control axon guidance (Fu et al., 2013). Thus, polyglutamylation could increase DCX and MAP1B binding to stabilize microtubules in the axon and reduce axon retraction during development. Additionally, the increase in polyglutamylation and MAP localization in response to Netrin-1 could be important for microtubule intrusion and polymerization into the growth cone for tuned response to Netrin-1. Our data indicates that DCX may help stabilize the microtubule cytoskeleton in response to Netrin-1. Because DCX strengthens the microtubule lattice, its increase in the growth cone could be an important response to Netrin-1 stimulation. Additionally, DCX localization increases in the actin-rich protrusions of the growth cone, which could be an important aspect of Netrin-1 response (Tint et al., 2009; Fu et al., 2013). Similar to MAP1B, overexpression of TTLL1 prevents the increase in DCX following Netrin-1 stimulation in the axon. This may cause axonal microtubules to be less stable and reduce the ability for the axon to grow in response to Netrin-1. TTLL1 regulation is important for proper Netrin-1 response. An overabundance of the protein may cause problems with tuning the levels of polyglutamylation and therefore there is a dampened response to Netrin-1 stimulation.

Our study shows that Netrin-1 increases microtubule polyglutamylation which is required for axons to grow more quickly. TTLL1 is required for the axon growth response to Netrin-1. However, increased levels of TTLL1 also inhibit the effects of Netrin-1 on growth. These data suggest that tight control of TTLL1 is important for axon response to Netrin-1 due to its role in extending glutamate chains on microtubules, which can lead to MAP binding and stabilization of the microtubule lattice.

## Data Availability

The original contributions presented in the study are included in the article/Supplementary material, further inquiries can be directed to the corresponding author.

## Supplementary material

SUPPLEMENTARY FIGURE S1 Netrin-1 was expressed in Cos-7 cells and purified for addition to cultured neurons.

SUPPLEMENTARY FIGURE S2 TTLL1 shRNA abolishes Netrin-1 induced increase in GFP-CSAP. GFP-CSAP fluorescence decays over time due to photobleaching. The decay of fluorescence signal significantly slows after addition of Netrin-1 in the control neurons **(A–C)** whereas decay does not change after addition of Netrin-1 in TTLL1 knockdown neurons **(C–F)**. GFP-CSAP does not significantly increase with Netrin-1 in TTLL1 overexpressing neurons **(G,H)**. GFP-CSAP fluorescence was not visible in scramble control neurons at the same laser power (data not shown).

SUPPLEMENTARY FIGURE S3 Neurons overexpressing TTLL1 have significantly more axonal branches than control neurons.
