# Supplementary material for: Netrin-1 stimulated axon growth requires the polyglutamylase TTLL1
Source: Front Neurosci. 2024 Oct 14;18:1436312. doi: 10.3389/fnins.2024.1436312 (PMC11514365; doi:10.3389/fnins.2024.1436312)
Supplement: SUPPLEMENTARY FIGURE S1 — Netrin-1 was expressed in Cos-7 cells and purified for addition to cultured neurons. [file Image_1.pdf]

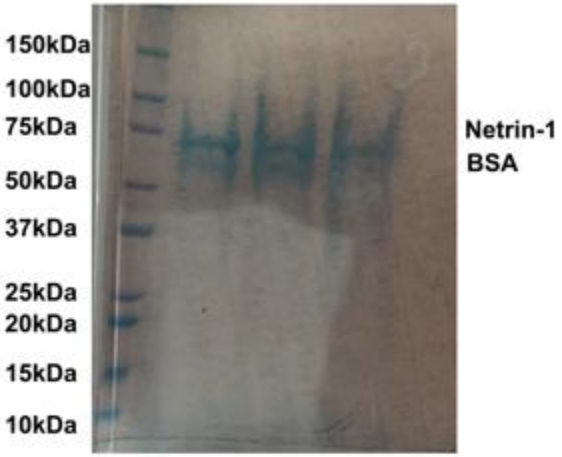


Supplemental Figure 1
Netrin-1 was expressed in Cos-7 cells and purified for addition to cultured neurons
