# Supplementary figures and images for: Netrin-1 stimulated axon growth requires the polyglutamylase TTLL1
Source: Front Neurosci. 2024 Oct 14;18:1436312. doi: 10.3389/fnins.2024.1436312 (PMC11514365; doi:10.3389/fnins.2024.1436312)

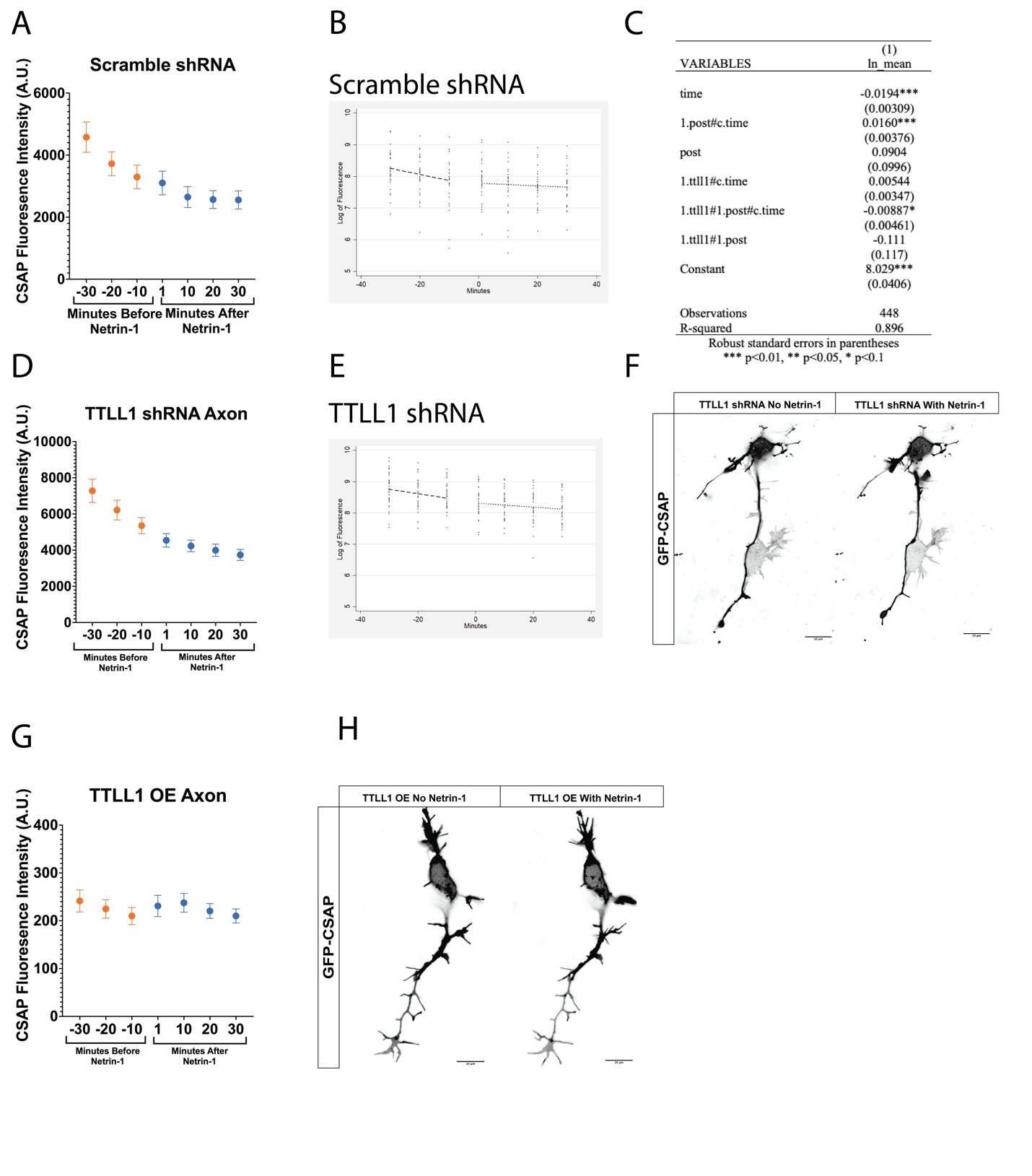

Supplement: SUPPLEMENTARY FIGURE S2 — TTLL1 shRNA abolishes Netrin-1 induced increase in GFP-CSAP. GFP-CSAP fluorescence decays over time due to photobleaching. The decay of fluorescence signal significantly slows after addition of Netrin-1 in the control neurons (A–C) whereas decay does not change after addition of Netrin-1 in TTLL1 knockdown neurons (C–F). GFP-CSAP does not significantly increase with Netrin-1 in TTLL1 overexpressing neurons (G,H). GFP-CSAP fluorescence was not visible in scramble control neurons at the same laser power (data not shown). [file Image_2.png]

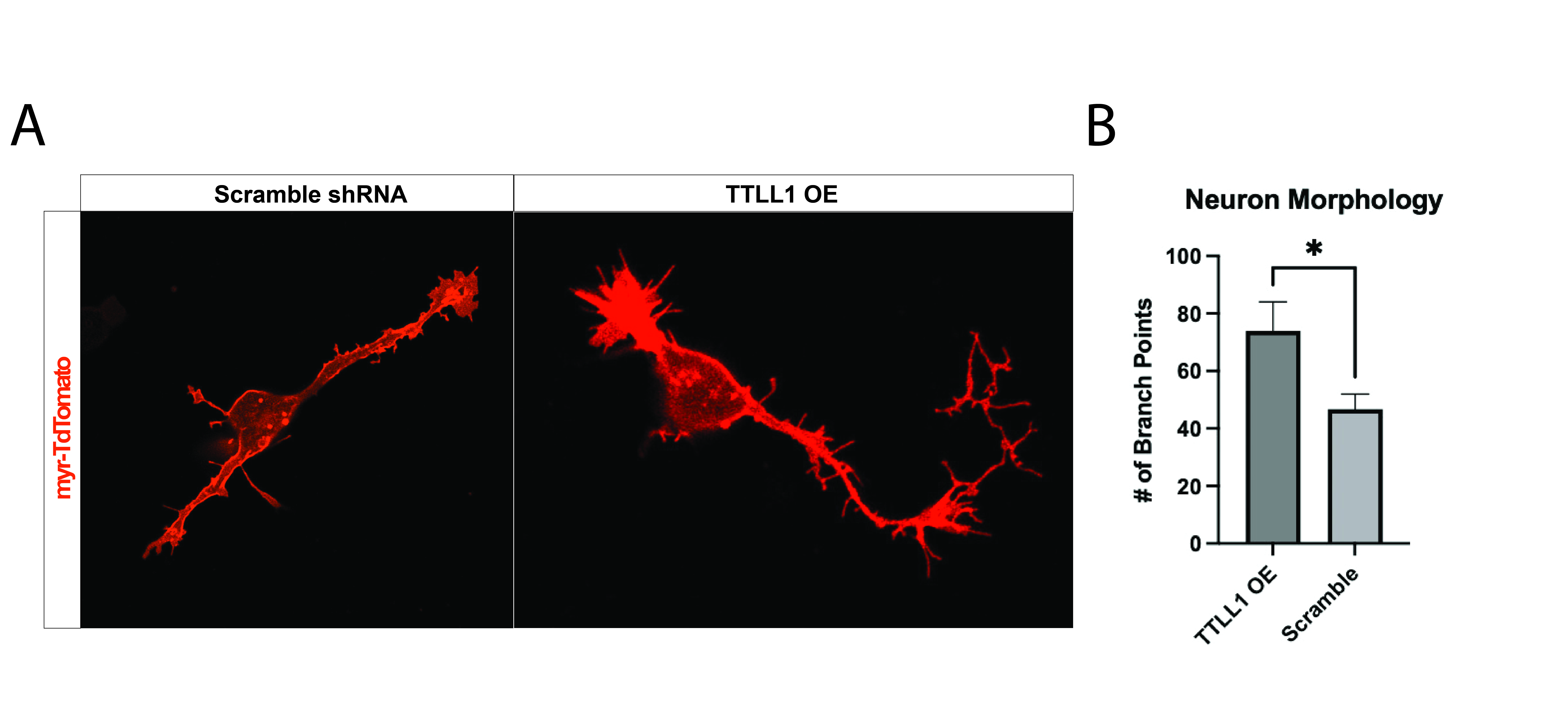

Supplement: SUPPLEMENTARY FIGURE S3 — Neurons overexpressing TTLL1 have significantly more axonal branches than control neurons. [file Image_3.jpg]
